# Lymphoepithelial cyst of the pancreatic tail: a rare entity with surgical and histopathological insights

**DOI:** 10.1093/jscr/rjaf186

**Published:** 2025-03-20

**Authors:** William T Rumble, Priscilla Martin

**Affiliations:** Department of General Surgery, Sunshine Coast University Hospital, Birtinya, 4575 Sunshine Coast, Australia; Department of General Surgery, Sunshine Coast University Hospital, Birtinya, 4575 Sunshine Coast, Australia

**Keywords:** pancreatic cystic neoplasm, lymphoepithelial cyst, pancreatic tail lesion, laparoscopic distal pancreatectomy

## Abstract

Lymphoepithelial cysts (LECs) of the pancreas are rare, benign lesions often discovered incidentally during imaging for unrelated conditions. Their radiological features frequently overlap with those of other more common pancreatic cystic lesions, presenting a diagnostic challenge. In this report from a general surgical department on the Australia, we detail the assessment and management of a 54-year-old male with an enlarging pancreatic tail lesion initially thought to be a mucinous cystic neoplasm. Laparoscopic distal pancreatectomy and splenectomy was performed, and subsequent histopathological evaluation confirmed a benign LEC. This report reviews the literature base, clinical presentation, radiological findings, surgical approach, and pathological diagnosis of this rare condition. We emphasize the importance of a tailored approach to management and the role of surgery in definitive treatment.

## Introduction

Pancreatic cystic neoplasms (PCN) represent a challenging and diverse group of pathological entities ranging from benign to malignant. The growing use of advanced imaging has led to increased detection of incidental pancreatic cysts, estimated to occur in 2%–20% of imaging studies [1]. Numerous guidelines have been developed to assist clinicians deciding which cysts to ignore, monitor, or resect. These include the Kyoto guidelines, American College of Gastroenterology guidelines, and European guidelines [2–4]. Their criteria and recommendations differ, but all incorporate assessment of clinical presentation, tumour markers, cross-sectional imaging and endoscopic ultrasound and fine needle aspiration (EUS-FNA) [5]. Unfortunately, no single treatment algorithm is perfect and the diversity of aetiologies for PCN means some cases remain unclear despite rigorous workup. As a result, surgery is often required for definitive diagnosis and management [6].

Among the causes of PCN, lymphoepithelial cysts (LECs) are one of the rarest. They are a benign lesion that comprise ˂0.5% of PCNs [7]. Like most PCNs, LECs are typically discovered incidentally; however, their imaging characteristics often overlap with pre-malignant cystic lesions, such as mucinous cystadenomas or intraductal papillary mucinous neoplasm. The literature on LECs is limited to case studies and series [6–13]. Efforts have been made to identify clinical and radiological features that can distinguish LECs from malignancies [10], however none can currently be used.

This case report highlights the diagnostic challenges and surgical management of a pancreatic tail cystic lesion in a 54-year-old male. The lesion ultimately proves to be a lymphoepithelial cyst of the pancreas, contributing to the growing body of literature on this rare entity.

## Case report

A 54-year-old male was referred in February 2022 to the University Hospital general surgical clinic for evaluation of an incidentally found pancreatic cystic lesion. The lesion was detected during a computed tomography (CT) scan performed by the GP to investigate renal colic.

His medical history included hypertension, gout and renal calculi. He denied previous episodes of pancreatitis or previous abdominal surgeries. His only medication was allopurinol. His blood analysis was all within normal limits, including his lipase of 40 U/L (normal range <180 U/L) and CA 19–9 of 33 U/mL (normal range 0–37 U/mL).

**Figure 1:**
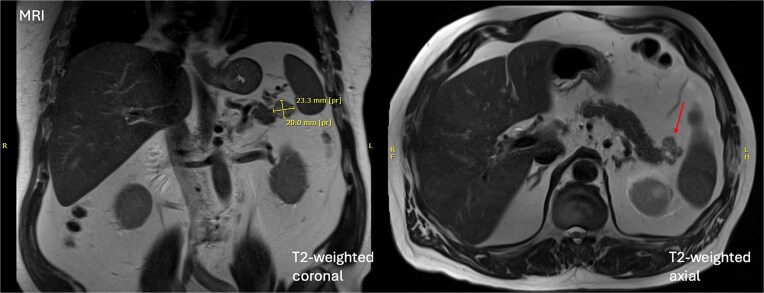
Initial MRI demonstrating a 20 × 26 × 20 mm mixed solid and cystic lesion in the pancreatic tail.

At the time of review, the patient was asymptomatic. Physical examination was unremarkable. A dedicated magnetic resonance imaging (MRI) demonstrated a 20 × 26 × 20 mm mixed solid and cystic lesion in the pancreatic tail with T1 hyperintensity, consistent with proteinaceous contents, but no interval growth since earlier imaging in 2020 (illustrated in Fig. 1). This stability favoured a benign aetiology, with pseudocyst or low-grade cystic neoplasm being the primary considerations. EUS-FNA was deemed unfeasible given the cyst’s location, and instead serial MRI imaging was arranged as per the Kyoto guidelines [2].

**Figure 2:**
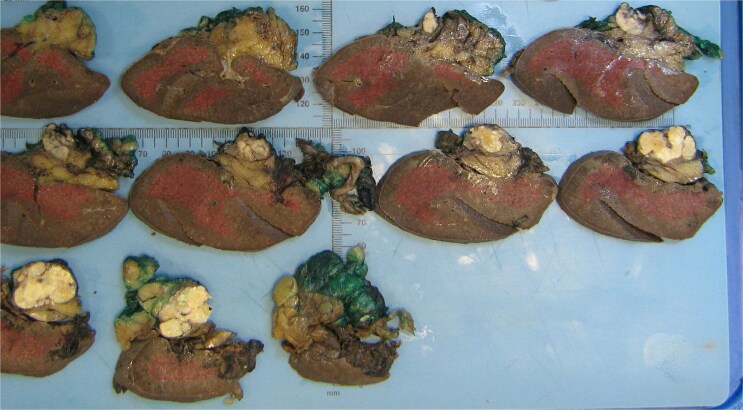
Macroscopic analysis of the operative specimen demonstrating a cystic lesion in the tail of the pancreas containing caseous (cheese-like) material.

A follow-up MRI in July 2024 demonstrated interval enlargement to a maximal diameter of 31 mm, and new septal enhancement. Again, no pancreatic ductal dilation or other masses were identified. Given the lesion now possessed three worrisome features—diameter >3 cm, septal enhancement and growth rate >2.5 mm per year—the decision was made to proceed with surgical resection.

The patient underwent a laparoscopic distal pancreatectomy. The lesion was intraoperatively abutting the hilum of the spleen. There was no local or distant spread of disease. Splenic artery and vein dissection revealed no plane allowing safe preservation of the spleen, prompting the decision to proceed with splenectomy. The operation was otherwise uncomplicated**.**

The patient recovered uneventfully. He was discharged on postoperative day 3 on a full diet. Follow-up in November 2024 revealed no ongoing abdominal symptoms.

**Figure 3:**
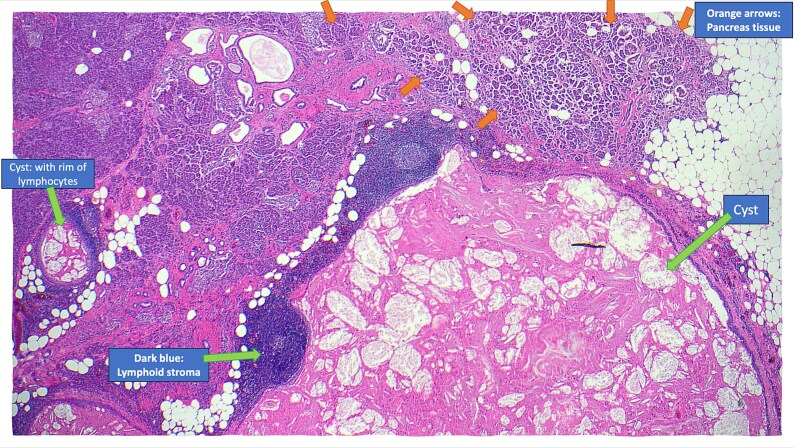
Annotated haematoxylin and eosin-stained microscopic cross-sectional sample demonstrating atrophic pancreatic parenchyma adjacent to the multilocular cyst with benign lymphoid stroma and reactive lymphoid follicles.

**Figure 4:**
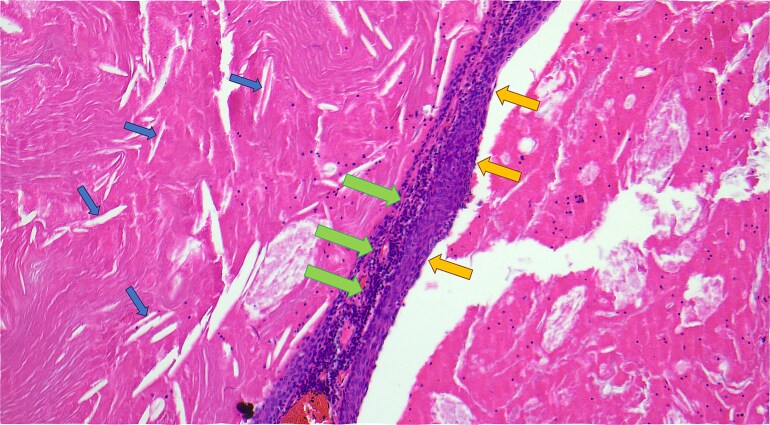
Haematoxylin and eosin-stained microscopic cross-sectional sample demonstrating numerous cholesterol clefts (small left arrows), a band-like rim of lymphocytes (middle large arrows), and the squamous epithelial lining of the cyst wall (medium right arrows).

The resected specimen is shown in Fig. 2 and included the distal pancreas and spleen, weighing 202.2 g in total. The pancreas contained a well-circumscribed, lobulated cystic lesion measuring 37 × 27 × 19 mm. The lesion was adjacent to the splenic hilum but did not infiltrate the spleen. The cut surface of the lesion showed multiloculated cystic spaces with tan, necrotic-appearing areas. Histopathological analysis is illustrated in Figs 3 and 4, which confirmed a benign lymphoepithelial cyst. The lesion was clear of all surgical margins, and there was no evidence of malignancy in peri-pancreatic lymph nodes or surrounding tissues.

## Discussion

Pancreatic lymphoepithelial cysts are rare entities with unique histopathological features. They were first described in the literature in 1985 [12]. The mean age of detection is 56 years old, and 80% are found in males [8]. They make up only 0.5% of resected pancreatic cysts, and have been identified in all parts of the pancreas as well as occasionally in extra-pancreatic location [7]. Most patients are asymptomatic, and the lesions are often incidentally discovered on imaging. However, particularly if the cyst is large, patients may present with abdominal pain. The pathogenesis of LECs is unclear but is thought to involve cystic transformation and squamous metaplasia of pancreatic ducts [8]. Radiologically, LECs often mimic more common cystic neoplasms, such as mucinous cystadenomas or pseudocysts. MRI and CT findings, including stable size, lack of enhancement, and absence of pancreatic duct involvement, can favour this diagnosis. However, definitive diagnosis requires histopathological examination, as was necessary in this case. The hallmark of LECs is in their microscopic features, which include unilocular or multilocular cystic structure lined by squamous epithelium, with a cyst wall containing lymphocyte rich cuff with germinal centres [14]. This distinct histology differentiates LECs from other cystic lesions, providing reassurance of their benign nature.

This case highlights the importance of a systematic approach to the management of pancreatic cystic lesions. While non-invasive diagnosis of this benign pancreatic lesion with FNA is clearly preferable, this is rarely possible. We suggest a future direction for research in this area is working to identify reliable pre-operative findings that can potentially negate the need for surgical excision. However, at present, and as in this case, LECs almost invariably require surgical intervention for definitive diagnosis due to their radiological similarities to other pre-malignant and malignant lesions. This case underscores the importance of a multidisciplinary approach, combining advanced imaging, surgical expertise, and detailed histopathological analysis, to optimize patient outcomes.
